# Laboratory assessment of rivaroxaban: a review

**DOI:** 10.1186/1477-9560-11-11

**Published:** 2013-07-03

**Authors:** Meyer Michel Samama, Geneviève Contant, Theodore E Spiro, Elisabeth Perzborn, Lena Le Flem, Céline Guinet, Yves Gourmelin, Gabriele Rohde, Jean-Luc Martinoli

**Affiliations:** 1 Hôtel-Dieu University Hospital, 1 place du Paris Notre-Dame, Paris 75004, 4ème, Paris, France; 2 Biomnis Laboratories R & D, 78 avenue de Verdun, BP 110,94200 Ivry-sur-Seine, Paris, France; 3 Diagnostica Stago SA, 125 avenue Louis Roche, P.A.E. Parispace 3, 92230 Gennevilliers, France; 4 Bayer HealthCare Pharmaceuticals Inc., PO Box 1000, 07045-1000 Montville, NJ, USA; 5 Bayer HealthCare AG, Aprather Weg 18a, D-42096 Wuppertal, Germany

**Keywords:** Factor Xa, Laboratory assessment, Rivaroxaban

## Abstract

Research into new anticoagulants for preventing and treating thromboembolic disorders has focused on targeting single enzymes in the coagulation cascade, particularly Factor Xa and thrombin, inhibition of which greatly decreases thrombin generation. Based on the results of phase III clinical trials, rivaroxaban, a direct Factor Xa inhibitor, has been approved in many countries for the management of several thromboembolic disorders. Owing to its predictable pharmacokinetic and pharmacodynamic characteristics, fixed-dose regimens are used without the need for routine coagulation monitoring. In situations where assessment of rivaroxaban exposure may be helpful, anti-Factor Xa chromogenic assays (in tandem with standard calibration curves generated with the use of rivaroxaban calibrators and controls) could be used. It is important to note that test results will be affected by the timing of blood sampling after rivaroxaban intake. In addition, the anti-Factor Xa method measures the drug concentration and not the intensity of the drug’s anticoagulant activity, and a higher than expected rivaroxaban plasma level does not necessarily indicate an increased risk of bleeding complications. Therefore, clinicians need to consider test results in relation to the pharmacokinetics of rivaroxaban and other patient risk factors associated with bleeding.

## Introduction

Traditional anticoagulant agents such as vitamin K antagonists (VKAs), unfractionated heparin (UFH), low molecular weight heparins and fondaparinux have been widely used in the prevention and treatment of thromboembolic diseases. However, these agents are associated with limitations, such as the need for regular coagulation monitoring (VKAs and UFH) [1,2] or a parenteral route of administration (UFH, low molecular weight heparin and fondaparinux) [2]. These limitations have prompted the development of target-specific oral anticoagulants that directly inhibit single enzymes in the coagulation pathway, such as Factor Xa or thrombin. Two direct Factor Xa inhibitors (rivaroxaban [Xarelto®, Bayer Pharma AG and Janssen Pharmaceuticals, Inc.] and apixaban [Eliquis®, Bristol-Myers Squibb and Pfizer EEIG]) and a direct thrombin inhibitor (dabigatran etexilate [Pradaxa®, Boehringer Ingelheim International GmbH]) are approved in many countries for the prevention of venous thromboembolism (VTE) after elective hip or knee replacement surgery in adults, and in the European Union (EU) and North America for the prevention of stroke and systemic embolism in adult patients with non-valvular atrial fibrillation [3-8]. In addition, rivaroxaban is also approved in the EU and North America for the treatment of deep vein thrombosis (DVT) and pulmonary embolism, and prevention of recurrent DVT and pulmonary embolism in adults, and is now approved in the EU, in combination with antiplatelet agents, for the prevention of atherothrombotic events in adults who have acute coronary syndromes and elevated cardiac biomarkers [3,4,9].

The mechanisms of action of anticoagulant agents have an important role in the prolongation of clotting time in tests such as the prothrombin time (PT) test. VKAs interfere with the γ­carboxylation of glutamate residues in Factors II, VII, IX and X, with the result that the coagulant activity of these factors is diminished. Direct Factor Xa inhibitors limit thrombogenesis via selective inhibition of Factor Xa without requiring cofactors such as antithrombin [10]. Direct thrombin inhibitors target thrombin and, likewise, do not require cofactors such as antithrombin [11]. Both classes of anticoagulant agents have predictable, dose-dependent anticoagulant effects [12]. Rivaroxaban inhibits free Factor Xa and prothrombinase activity as well as clot-bound Factor Xa, thus effectively blocking thrombin generation [13]. Inhibition of Factor Xa activity by rivaroxaban is closely correlated to its plasma concentration. Anti-Factor Xa activity can be measured to indicate rivaroxaban exposure.

As with apixaban and dabigatran, rivaroxaban does not require routine coagulation monitoring or dose titration (unlike VKAs and UFH). However, a reliable laboratory assay that could measure exposure to rivaroxaban may be necessary or helpful in certain clinical circumstances (e.g. prior to urgent surgery, for perioperative management of those receiving rivaroxaban, for patients with thromboembolic or bleeding events, or for suspected overdose). Because rivaroxaban and other target-specific oral anticoagulants have different mechanisms of action from traditional anticoagulant agents, laboratory tests used for these traditional agents (such as PT/international normalised ratio [INR] or activated partial thromboplastin time) are not suitable for target-specific oral anticoagulants [14]. This article will summarise the pharmacokinetics and pharmacodynamics of rivaroxaban and provide information and guidance on laboratory tests that can be used for the measurement of rivaroxaban in clinical practice.

## Pharmacokinetics and pharmacodynamics of rivaroxaban

The oral bioavailability of rivaroxaban is 80–100% for the 10 mg dose, irrespective of food intake [3,15]. Under fed conditions, rivaroxaban 10 mg, 15 mg and 20 mg tablets demonstrate dose-proportional bioavailability. In a fasted state, rivaroxaban pharmacokinetics are approximately linear up to about 15 mg once daily, and oral bioavailability is reduced to 66% after a 20 mg tablet; at higher doses, bioavailability decreases as a result of poor solubility [3,16]. Food does not affect the area under the concentration–time curve or maximum plasma concentration (C_max_) of the 10 mg dose [3]. The administered oral dose of rivaroxaban is absorbed rapidly, with C_max_ occurring 2–4 hours after tablet intake [15].

At total daily oral doses of rivaroxaban of 5–60 mg, C_max_ ranges (mean values) from 40 μg/l to 400 μg/l, and minimum plasma concentration (C_trough_) (mean values) from 8 μg/l to 160 μg/l (data derived from phase II studies of rivaroxaban in patients undergoing hip replacement surgery, patients with DVT or patients with acute coronary syndrome; Table 1) [17-19].

**Table 1:** Summary of pharmacokinetic characteristics of rivaroxaban at steady state based on phase II data

**Parameter**	**Rivaroxaban dose**					
	2.5 mg bid*	**2.5 mg bid**^**#**^	5 mg bid*	10 mg bid*	20 mg bid*	**30 mg bid**^**‡**^
AUC (μg.h/l)	300	328	464	974	1764	6500
C_max_ (μg/l)	53	40	86	180	299	400
C_trough_ (μg/l)	8	14	11	38	68	160

*Data are mean values from an analysis of the ODIXa-HIP2 study [17]. ^#^Data are median values from an analysis of the ATLAS ACS TIMI 46 study (subset of patients aged <50 years) [18]. ^‡^Data are mean values from an analysis of the ODIXa-DVT study [19].

Abbreviations: *AUC* area under the concentration–time curve, *bid* twice daily, *C*_*max*_ maximum plasma concentration, *C*_*trough*_ minimum plasma concentration.

No relevant accumulation occurs beyond steady state in healthy individuals [20]. Elimination of rivaroxaban from plasma occurs with a terminal half-life of 5–9 hours in young individuals [3,20] and 11–13 hours in the elderly [3,21]. Rivaroxaban has a dual mode of elimination. Of the administered dose, approximately two-thirds undergoes metabolic degradation, half of which is eliminated renally and the other half by the hepatobiliary route. The final one-third of the administered dose undergoes direct renal excretion as unchanged active substance in the urine, mainly via active renal secretion. Rivaroxaban has no major or active circulating metabolites [3,22].

In phase I studies in healthy subjects [15,20,23] and in phase II studies of patients undergoing major orthopaedic surgery [17] or those with acute coronary syndrome [18], rivaroxaban was found to have predictable, dose-dependent pharmacokinetics. In addition, population modelling suggests that pharmacokinetic parameters are generally similar between different patient groups (i.e. VTE prevention and treatment of acute DVT) [19].

## Influence on coagulation assays

### Prothrombin time

Rivaroxaban prolongs PT (measured in seconds or PT ratio) in a linear and concentration-dependent manner when using reagents sensitive to rivaroxaban, such as Neoplastin Plus® (Diagnostica Stago, Asnières-sur-Seine, France) or HemosIL RecombiPlasTin 2G (Instrumentation Laboratory, Bedford, MA, USA). However, results vary according to the thromboplastin reagent used, as shown by *in vitro*[13,24-26] and *ex vivo* studies in patients undergoing hip or knee replacement surgery (total daily doses of 5–60 mg) [17]. The concentration of rivaroxaban required to double PT in human plasma using Neoplastin Plus is 301 μg/l [13]. In contrast, the concentration of rivaroxaban required to double PT in human plasma with Innovin® (Siemens HealthCare Diagnostics, Marburg, Germany) is 700 μg/l [24]. Neoplastin Plus can be used in a dilute PT test, which is more sensitive than the PT test [24]. As with the PT test, rivaroxaban results in a linear concentration-dependent prolongation of dilute PT, with varying gradients of the concentration–effect relationships for different thromboplastin reagents, as shown by an *in vitro* study [24].

### Other coagulation assays

Rivaroxaban prolongs clotting times concentration-dependently in the activated partial thromboplastin time [24], HepTest (Sekisui Diagnostics, Stamford, CT, USA [27]) [15,20] and prothrombinase-induced clotting time (PiCT) test [24,26,28]. However, for the HepTest and PiCT test, there is a paradoxical shortening of clotting time at low rivaroxaban concentrations when bovine Factor Xa is used [24]. This effect is not seen with a shorter or no incubation period, when antithrombin-depleted (immunodeficient) plasma (instead of platelet-poor plasma) is used [24,28], or when human Factor Xa is used (in the PiCT test only) [28]. Rivaroxaban also affects thrombin generation parameters (prolonging lag time and time to peak of thrombin generation) and decreases peak thrombin generation and endogenous thrombin potential [24,29]. There is a more pronounced effect on the initiation and propagation phases of thrombin generation than on the decay phase [30]. However, thrombin generation tests are not available in many clinical laboratories.

### Inhibition of Factor Xa activity

Inhibition of Factor Xa and plasma rivaroxaban levels are closely correlated [20]. Therefore, assays (particularly chromogenic assays) that measure inhibition of Factor Xa activity can quantify rivaroxaban plasma concentrations [25,26,31-33]. These types of assay are able to measure a wide range of rivaroxaban concentrations (e.g. 20–500 μg/l) by use of a reference calibration curve for rivaroxaban spiked in plasma [31]. Standardisation of these assays to measure rivaroxaban involves the use of rivaroxaban calibrators and controls [33], and standardised assay kits are now commercially available for clinical use (e.g. BIOPHEN Factor X Chromogenic [Aniara, West Chester, OH, USA], STA® Liquid Anti-Xa and STA® rivaroxaban calibrator and control [Diagnostica Stago], Technochrom anti-Xa and Technoview rivaroxaban calibrator and control [Technoclone GmbH, Vienna, Austria]).

## Is prothrombin time suitable for measuring rivaroxaban?

The variability in responses between thromboplastin reagents used in the PT test is too large when the results are expressed in seconds for samples containing the same concentrations of rivaroxaban [24,34]. This variation is caused by different sensitivities of the reagents to rivaroxaban [34], possibly caused by interactions between Factor Xa inhibitors and phospholipids in thromboplastin reagents [35]. This variability is not corrected by conversion of PT to INR values [24]. The INR has been developed specifically for monitoring anticoagulation with VKAs [1] and, therefore, should not be used for rivaroxaban. Moreover, conversion of PT to INR values can increase the variability [36].

Normal PT can indicate intact haemostatic function [37]. An *in vitro* study showed that reduction of the variability in PT results across thromboplastin reagents by use of an international sensitivity index valid for rivaroxaban is feasible [38]. In addition, a modified PT test could be potentially useful [24]. Recently, a study assessing rivaroxaban effect with Simplastin® Excel S reagent (Tcoag Ireland, County Wicklow, Ireland) suggested that it might have a similar effect to VKAs [39]. Results were reported in a field trial of 18 centres that compared PT results from different laboratories using different local reagents versus one central reagent and local instruments. This trial reported inter-laboratory variation in mean PT values that was significantly reduced by use of a centrally provided PT reagent (STA® Néoplastine CI Plus [Diagnostica Stago]) and when results were expressed as rivaroxaban concentrations (μg/l) [40]. This method may be sensitive enough to measure peak rivaroxaban plasma concentrations that would occur after currently approved therapeutic doses, i.e. provide a qualitative confirmation of the presence of rivaroxaban [37]. However, it lacks precision, particularly at low rivaroxaban concentrations and is, therefore, not suitable for measuring rivaroxaban levels in blood samples taken near the time of C_trough_[37]. In addition, specific calibrators for use with the PT test are not commercially available.

The effect of rivaroxaban (as with other target-specific oral anticoagulants) on PT is short-lived (e.g. minimal effect at 24 hours post-dosing [3]) and changes over time, whereas the effects of VKAs on PT last for several days [1,20,21]. In addition, the PT test, like other global clotting assays, is not specific for Factor Xa and can be influenced by many disease conditions, such as liver disease, some types of cancer and Hodgkin’s disease [41-43].

## Are anti-Factor Xa chromogenic assays the preferred method for measuring rivaroxaban?

Anti-Factor Xa chromogenic assays can accurately measure a wide range of rivaroxaban concentrations in plasma – i.e. give a quantitative measure of the rivaroxaban level – provided that a standard calibration curve is generated with rivaroxaban calibrators and controls [33,44,45]. A field trial of 23 centres has shown that anti-Factor Xa chromogenic assays, in conjunction with calibrators and controls for rivaroxaban, can measure rivaroxaban concentrations in plasma in the range of 20–660 μg/l [33]. Furthermore, the mean rivaroxaban concentrations measured were in agreement with the expected values, even at the low rivaroxaban concentration when a modified STA® Rotachrom® test (Diagnostica Stago) set-up was used. In this test, plasma samples were diluted to a ratio of 1:4 in Owren–Koller assay buffer to enable measurement of rivaroxaban concentrations >100 μg/l. Moreover, there was less variation among the different laboratories when using the centrally provided reagent compared with using local anti-Factor Xa reagents, particularly for the lowest rivaroxaban plasma concentration. The coefficients of variation at 20 μg/l reached 37.0% with local methods, compared with 19.1% with the centrally provided reagent; 13.7% versus 10.9% at 199 μg/l actual rivaroxaban value; and 14.1% versus 10.0% at 662 μg/l actual rivaroxaban value (1:3 diluted plasma). In addition, a Swiss study across nine laboratories showed that inter-laboratory precision of the chromogenic anti-Factor Xa assay, Biophen Heparin 6 (Hyphen Biomed, Neuilly-sur-Oise, France), was satisfactory, with the coefficient of variation in the range of 2.6–10.5% [45].

Another study measured rivaroxaban in *ex vivo* blood samples from patients who received rivaroxaban 10 mg once daily for VTE prevention after hip or knee replacement surgery [44]. With the use of rivaroxaban calibrators and controls (set to enable measurement of plasma rivaroxaban ≥10 μg/l) in this study, three chromogenic anti-Factor Xa methods were evaluated: one with the addition of exogenous antithrombin (Technochrom® anti-Xa [AT+] [Technoclone, Vienna, Austria]) and two without the addition of antithrombin (COAMATIC® Heparin [Chromogenix, Milan, Italy]; and Technochrom® anti-Xa, [Technoclone]). All of the assays showed a linear relationship between the optimal density of the chromogenic assays and rivaroxaban concentrations validated by high-performance liquid chromatography coupled with tandem mass spectrometry. However, the method in which antithrombin was added detected falsely high rivaroxaban levels [44]. These findings support the suitability of anti-Factor Xa assays (without the addition of antithrombin) in measuring rivaroxaban concentrations. Although anti-Factor Xa chromogenic assays differ in their sensitivity to rivaroxaban, mathematical modelling could decrease the variation between assays [32]. Anti-Factor Xa chromogenic assay kits specifically developed for rivaroxaban calibrators and controls are now commercially available for clinical use.

### Interpretation of measured plasma levels

An understanding of expected rivaroxaban plasma concentrations after therapeutic doses is important for the interpretation of measured results. The plasma levels of rivaroxaban in phase II studies are shown in Table 2[18,19,46].

**Table 2:** Rivaroxaban plasma concentrations after therapeutic doses based on phase II data and simulated virtual data

**Dose**	**Clinical setting**	**C**_**trough **_**(μg/l)**	**C**_**max **_**(μg/l)**
2.5 mg bid	Acute coronary syndrome	16 (6–34)*	44 (28–66)*
10 mg od	VTE prevention after total hip replacement	9 (1–38)^#^	125 (91–196)^#^
15 mg od	Stroke prevention in patients with AF (CrCl ≤50 ml/min)	57 (18–136)^‡^	229 (178–313)^‡^
20 mg od	DVT treatment (continued treatment)	26 (6–87)^§^	270 (189–419)^§^
20 mg od	Stroke prevention in patients with AF (CrCl >50 ml/min)	44 (12–137)^‡^	249 (184–343)^‡^

*Estimated parameters at steady state – median values (5th–95th percentile range) [18]. ^#^Estimated parameters at steady state – median values (5th–95th percentile range) in patients undergoing hip replacement surgery [46]. ^‡^Estimated parameters at steady state – geometric means (5th–95th percentile range) in stroke prevention in patients with AF (Bayer HealthCare Pharmaceuticals and Janssen Research & Development, LLC: data on file). ^§^Estimated parameters at steady state – geometric means (5th–95th percentile range) in phase II studies in the acute treatment of DVT [19].

Abbreviations: *AF* atrial fibrillation, bid: twice daily, *C*_*max*_ maximum plasma concentration, *CrCl* creatinine clearance, *C*_*trough*_ minimum plasma concentration, *DVT* deep vein thrombosis, *od* once daily, *VTE* venous thromboembolism.

In addition, data show that there is some inter-individual variability in plasma rivaroxaban concentrations, but the clinical relevance of this variation has not been determined. In healthy individuals, approximate geometric coefficients of variation for C_max_ are 16% after a 10 mg dose, 36% after a 20 mg dose [15] and 19% after a 20 mg twice-daily dose [20]. However, after hip replacement surgery, the geometric coefficients of variation of rivaroxaban at total daily doses of 5–20 mg are higher than in healthy individuals and in the range of 60–93% for C_trough_ and 47–74% for C_max_[17].

Accumulating data indicate that anti-Factor Xa chromogenic assays (with use of rivaroxaban calibrators and controls) are able to measure a wide range of rivaroxaban concentrations encompassing the entire range after therapeutic dosing [1,2,24]. The timing of blood sampling after tablet intake is important because levels of rivaroxaban change markedly over time owing to the pharmacokinetics of the drug (e.g. levels of rivaroxaban will differ greatly 2–4 hours versus 24 hours after dosing).

## Conclusions

Unlike VKAs, the target-specific oral anticoagulants (such as rivaroxaban, dabigatran, apixaban and edoxaban) are currently used in clinical practice at fixed doses without the need for routine coagulation monitoring [47]. The concentration of target-specific oral anticoagulants may potentially need to be measured in certain clinical situations [48], such as before urgent surgery, perioperative management, thromboembolic or bleeding events, or in cases of suspected overdose. The conventional PT/INR method has some important limitations. For measuring apixaban, anti-Factor Xa chromogenic assays are preferable to the PT test because they yield more accurate results [25]. Similarly, accumulating data indicate that the anti-Factor Xa chromogenic assay is the most suitable assay for the quantitative assessment of rivaroxaban, provided rivaroxaban calibrators and controls are used, and results are expressed as rivaroxaban concentration (μg/l) [33]. If this method is not available or in an emergency situation, such as before urgent surgery, the PT assay (expressed in seconds) using a thromboplastin reagent sensitive to rivaroxaban may be useful to indicate whether the anticoagulant effect of rivaroxaban is present [3], provided that the patient's baseline PT is not abnormal. However, interpretation of the results should take into account the pharmacokinetic characteristics of rivaroxaban. A PT reagent has recently been found to have particularly high sensitivity to the effects of rivaroxaban, but no explanation has been proposed [39]. A point-of-care assay has been studied for the qualitative assessment of rivaroxaban in urine samples [49], which may be potentially useful in investigating treatment compliance, for example. However, this method needs further validation if it is to be used in clinical practice.

Practical considerations for the quantitative measurement of rivaroxaban using anti-Factor Xa chromogenic assays include the timing of blood sampling, interpretation of test results and standardisation of the assays. The timing of blood sampling in relation to the pharmacokinetic characteristics of rivaroxaban is important because it will directly influence the test results; for example, plasma levels of rivaroxaban will be high in the period 2–4 hours after drug administration compared with 12–24 hours after dosing. Therefore, an understanding of the expected time to C_max_ and C_trough_ of rivaroxaban is important when interpreting test results. Another practical consideration is the interpretation of test results and potential clinical implications. It is important to note that the anti-Factor Xa method measures the drug concentration (quantitative assessment) and not the intensity of the drug’s anticoagulant activity (qualitative assessment) [24], whereas coagulation assays used for monitoring the traditional anticoagulants, VKAs and UFH, indicate the intensity of anticoagulation [1,2]. In addition, a higher than expected plasma level does not necessarily indicate an increased risk of bleeding complications but could signal a need for closer surveillance for risk of bleeding.

Standardised anti-Factor Xa chromogenic assays with rivaroxaban calibrators and controls (e.g. BIOPHEN Factor X Chromogenic [Aniara], STA® Liquid anti-Xa and STA® rivaroxaban calibrator and control [Diagnostica Stago], Technochrom anti-Xa and Technoview rivaroxaban calibrator and control [Technoclone]) are now commercially available for clinical use. These assays differ from the INR test and other clotting tests that have been used to monitor traditional anticoagulant agents in that the results are expressed as rivaroxaban concentrations (μg/l).

The perioperative management of patients receiving anticoagulants is an important issue, although guidelines on this are scarce. The principles of when to interrupt and resume rivaroxaban are based on the relatively short half-life, rapid onset of action and dual route of elimination of rivaroxaban [50,51]. The current consensus is that the last dose of rivaroxaban should be taken not less than 24 hours before elective surgery [52], and the manufacturer recommends resumption of rivaroxaban 6–10 hours after surgery, provided haemostasis has been established [3].

Based on the consistent efficacy and safety profiles demonstrated in the large-scale phase III clinical trial programme, fixed-dose regimens of rivaroxaban have been approved for clinical use in several indications. Routine measurement of rivaroxaban plasma levels or its pharmacodynamic effects is not required or recommended. Clinicians should adhere to the regulatory recommendations or the label, particularly in patients or clinical situations associated with an increased risk of bleeding. At present, physicians have many questions on the practical aspects of the use of target-specific oral anticoagulants in clinical practice [53], and detailed guidelines are lacking. For example, there is concern among clinicians about switching between anticoagulants and reversing anticoagulant effects; therefore, more recommendations are needed, although the respective labels of the newer agents provide some guidance on these issues [3-8]. To conclude, the choice of laboratory test for rivaroxaban will depend on the clinical situation: if a qualitative assessment of the presence of rivaroxaban in the blood is needed, the PT test is suitable provided that a rivaroxaban-sensitive reagent is used, whereas if a quantitative measurement of plasma rivaroxaban is required, an anti-Factor Xa chromogenic assay in tandem with rivaroxaban calibrators and controls with results expressed as rivaroxaban concentration (μg/l) can provide accurate results (Table 3). Whichever test is used, interpretation of results must take into account the timing of blood sampling (making reference to the pharmacokinetics of rivaroxaban) and differences in the functionality of the assays (qualitative versus quantitative).

**Table 3:** Suitable laboratory tests for rivaroxaban and clinical situations

**Chromogenic anti-Factor Xa assay**	**Prothrombin time test**
**(with rivaroxaban calibrators)**	**(with rivaroxaban-sensitive reagent)**
• Acute renal failure	• Emergency situations when an anti-Factor Xa assay is not available
• Bleeding complications or thrombosis during treatment	
• Emergency situations (if available in time):	
Prior to urgent surgery	
Life-threatening bleeding	
Stroke (if fibrinolytic treatment indicated)	
Acute percutaneous coronary intervention (if heparin bolus indicated)	
• Overdose	
• Suspected accumulation of drug	

## Abbreviations

AF: Atrial fibrillation; AUC: Area under the concentration–time curve; Bid: Twice daily; Cmax: Maximum plasma concentration; CrCl: Creatinine clearance; Ctrough: Minimum plasma concentration; DVT: Deep vein thrombosis; EU: European Union; INR: International normalised ratio; Od: Once daily; PiCT: Prothrombinase-induced clotting time; PT: Prothrombin time; UFH: Unfractionated heparin; US: United States; VKA: Vitamin K antagonist; VTE: Venous thromboembolism

## Competing interests

CG, LLF and MMS are employees of Biomnis Laboratories Clinical Research; they have no conflicts of interest. GC, YG and JLM are employees of Stago; they have no conflicts of interest. EP is a former employee of and consultant to Bayer HealthCare Pharmaceuticals (the manufacturers of rivaroxaban). TS is an employee of Bayer HealthCare Pharmaceuticals. GR is an employee of Bayer HealthCare AG.

## Authors’ contributions

MMS is the lead author. All authors contributed to the writing of the article. All authors read and approved the final manuscript.
